# Zika Virus Infection and Microcephaly: Evidence for a Causal Link

**DOI:** 10.3390/ijerph13101031

**Published:** 2016-10-20

**Authors:** Jin-Na Wang, Feng Ling

**Affiliations:** Zhejiang Provincial Center for Disease Control and Prevention, 3399 Binsheng Road, Hangzhou 310051, China; jnwang@cdc.zj.cn

**Keywords:** Zika virus (ZIKV), microcephaly, pregnancy, fetus

## Abstract

Zika virus (ZIKV) is a flavivirus related to the Dengue, yellow fever and West Nile viruses. Since the explosive outbreaks of ZIKV in Latin America in 2015, a sudden increase in the number of microcephaly cases has been observed in infants of women who were pregnant when they contracted the virus. The severity of this condition raises grave concerns, and extensive studies on the possible link between ZIKV infection and microcephaly have been conducted. There is substantial evidence suggesting that there is a causal link between ZIKV and microcephaly, however, future studies are warranted to solidify this association. To summarize the most recent evidence on this issue and provide perspectives for future studies, we reviewed the literature to identify existing evidence of the causal link between ZIKV infection and microcephaly within research related to the epidemics, laboratory diagnosis, and possible mechanisms.

## 1. Introduction

Zika virus (ZIKV), a mosquito-borne virus in the family Flaviviridae and genus *Flavivirus* [1], is mainly transmitted by many *Aedes* mosquitoes [2]. Besides, ZIKV can also be transmitted by placenta, transfusion, transplantation, sexual activity, etc. [3]. It was first isolated from a rhesus monkey in 1947, and it was not until 1952 that was first identified in humans [4]. In recent decades, outbreaks of ZIKV have occurred in areas ranging from Yap Island, Micronesia, in 2007 to French Polynesia in 2013 [5,6] and South America in 2015 [7]. As of 13 October 2016, 73 countries and territories have reported evidence of mosquito-borne ZIKV transmission since 2007, mainly distributed in Africa, America, Asia and the Pacific regions [8]. Furthermore, there were some countries had reported travel-associated transmission ZIKV cases, which gained increasing international attention.

ZIKV infection often causes mild symptoms, such as fever, skin rashes, headache, conjunctivitis, and muscle and joint pain, which usually last for two to seven days [9]. Interestingly, increasing evidence has indicated a possible causal link between ZIKV infection and adverse pregnancy outcomes, especially microcephaly. Microcephaly is a neonatal malformation defined as a head size much smaller compared with the normal size for the infant’s age and sex [10]. The etiology of microcephaly is complex and appears to be affected by multiple factors, including both genetic and environmental contributions, such as exposure to toxic chemicals, radiation, genetic abnormalities, severe malnutrition during fetal life, certain metabolic disorders, or infection in the womb such as toxoplasmosis, rubella, herpes, syphilis, cytomegalovirus and HIV, etc. [11,12]. However, West Nile virus is the only flavivirus previously reported to be associated with encephalitis resulting in fetal brain damage [13]. This review aims to summarize possible evidence of the causal link between ZIKV infection and microcephaly. 

## 2. Possible Evidence of the Causal Link between ZIKV Infection and Microcephaly

### 2.1. ZIKV Outbreak and the Subsequent Increase in Microcephaly Cases

In April 2015, a ZIKV outbreak began in Brazil [7]. The subsequent September, dramatic increases in the number of microcephaly cases were identified in Pernambuco State [14]. Then, the Brazil Ministry of Health released a report declaring an unusual increase in cases of microcephaly in Pernambuco [15] and suggesting an association with ZIKV infection [16]. On 30 November 2015, a microcephaly incidence 20 times greater than that observed in previous years was reported in Brazil [16]. On 1 February 2016, the Emergency Committee of the WHO declared that the recent cluster of microcephaly cases and other neurological disorders constituted a Public Health Emergency of International Concern (PHEIC) [17]. As of 22 June 2016, 12 countries and territories have reported cases of microcephaly and other central nervous system malformations potentially associated with ZIKV infection [8].

### 2.2. ZIKV Infection and Severe Birth Defects

Standard fetal ultrasound scans can detect microcephaly at early stages of development [18]. Symptoms of congenital ZIKV syndrome detectable by ultrasonography include brain atrophy, intracranial calcifications, dysgenesis of the corpus callosum, enlarged cisterna magna, and asymmetrical cerebral hemispheres [3]. One cohort study enrolled a total of 88 pregnant women, 72 of whom were positive for ZIKV infection. All enrolled women were healthy and without other risk factors for birth defects. Fetal ultrasonography was performed on 42 of the ZIKV-positive women and all of the 16 ZIKV-negative women. Following ultrasonography, fetal abnormalities, including intrauterine growth restriction, microcephaly, central nervous system (CNS) findings and fetal death, were detected in 29% of the 42 ZIKV-positive women but in none of the ZIKV-negative women [19], indicating ZIKV infection during pregnancy might cause adverse pregnancy outcomes. Computed tomography (CT) or magnetic resonance imaging (MRI) scans can also be used to identify symptoms of congenital ZIKV syndrome, including calcifications in the junction between cortical and subcortical white matter, malformations of cortical development, decreased brain volume, and ventriculomegaly [20]. Reverse transcription polymerase chain reaction (RT-PCR) has been identified as an effective method for ZIKV detection during the early stages of infection [21]. In December 2015, tissues samples from two newborns with microcephaly, who died within 20 h of birth, and products of conception from the miscarriages of two mothers who had clinical signs of ZIKV infection during pregnancy were tested. Among the samples from the newborns, only brain tissue tested ZIKV positive in the RT-PCR assay, while viral antigens were noted within the chorionic villi obtained from the products of conception [22]. Similar results were noted in two other case reports from February 2016 involving pregnant women with suspected ZIKV infection probably occurring at approximately 13 weeks of gestation. Small fetal heads and brain calcifications were identified in these two cases. After the autopsies, one infant had a large amount of viral genomic RNA present only in brain tissue, and the virus sequence isolated in this case was similar to that of other recently isolated ZIKV sequences [23]. In another case, the placenta, brain, lungs, heart, skin, spleen, thymus, liver, kidneys, and cerebral cortex of the fetus were tested for ZIKV RNA; however, only the brain sample was positive for ZIKV [24]. It should be mentioned that the findings of these case reports did not provide proof that ZIKV caused microcephaly or brain damage; however, to some extent, they strengthen the evidence of this link. Because ZIKV RNA had been identified in the brain tissue of infants and the placental tissue of miscarried fetuses whose mothers were infected with ZIKV during pregnancy, brain and early gestational placental tissue might be the preferred tissues for the viral attack. The successful isolation of ZIKV virus from fetal brain tissue fulfilled one of the Koch’s postulates and provided stronger evidence of this link. One cohort study enrolled 35 infants with microcephaly whose mothers reported residence in or travel during pregnancy to areas where ZIKV was circulating. Overall, 11 infants had redundant and excessive scalp skin. This study indicated that acute intrauterine brain injury affected cerebral growth but did not affect the growth of scalp skin [25], which also indicated a strong neurotropism of ZIKV. Driggers et al. described a case of a pregnant woman infected with ZIKV during the 11th gestational week. After serial fetal ultrasonography, they found that the fetal head circumference had decreased from the 47th percentile at 16 weeks of gestation to the 24th percentile at 20 weeks of gestation. In addition, they successfully isolated infectious ZIKV RNA from fetal brain tissue [26]. In this case, serial ultrasonography images showed a decrease in fetal head circumference, indicating that the rate of brain growth decreased after ZIKV infection. Successful isolation of ZIKV RNA from fetal brain tissue provided further evidence of this association. In addition to microcephaly, other congenital defects caused by ZIKV, such as placental damage and fetal demise, were also found, which suggested that microcephaly might be part of an overall syndrome of restricted fetal growth induced by maternal ZIKV infection. The strong neurotropism of ZIKV may not only lead to microcephaly of the fetus, but also Guillain-Barré syndrome (GBS) of the adults. ZIKV could be isolated from the cerebrospinal fluid of the GBS patients or indicated by immunologic findings, meaning that ZIKV might cause neurological complications [27]. Finally, on the basis of a review of the available evidence, and given Shepard’s and other criteria as a framework, Rasmussen et al. suggested a causal relationship existed between prenatal ZIKV infection and microcephaly [28]. This was the first establishment of a definite conclusion regarding the causal link between ZIKV and microcephaly. Given this recognition of a causal relationship, forthcoming research should demonstrate an increased effort to prevent and control ZIKV-related adverse outcomes.

### 2.3. Vertical Transmission

The earliest cases of perinatal ZIKV transmission were reported in December 2013 and February 2014 [29]; in these cases, transmission occurred around the time of delivery and caused mild disease in two newborns, indicating perinatal transmission of ZIKV might occur during delivery, breastfeeding, or exchange of saliva or other bodily fluids. Oliveira Melo et al. examined two pregnant women who were diagnosed with fetal microcephaly and suffered from symptoms related to ZIKV infection. Although both women’s blood samples were negative for ZIKV, amniocentesis and subsequent quantitative RT-PCR results were positive [13]. This might have been the first evidence of intrauterine transmission of ZIKV. There are several potential explanations for the negative maternal blood tests but positive fetal results. No gold standard has been indicated for the diagnosis of ZIKV, and the viremic period of ZIKV is believed to be short [30]. Although detection of viral RNA by RT-PCR has proven to be effective, it is thought to only be positive in serum four to seven days after symptom onset [21]. After that period, only negative results might be obtained. Similarly, RT-PCR tests may not be sensitive enough to detect ZIKV infection in a newborn who was infected in utero if the period of viremia had passed [31]. Additionally, a large proportion of patients with ZIKV infection have been asymptomatic [5], and these pregnant women did not appear to be severely affected by ZIKV infection [18]; therefore, a lack of symptoms does not negate a possibility of infection. These are all diagnostic problems that need to be solved in future studies. Adibi et al. proposed two hypotheses for why the virus can pass through the placenta and lead to teratogenic effects. The first was that the placenta directly conveys ZIKV to the embryo or fetus, and the second was that the effects were a result of the response of the placenta to virus exposure [32]. Noronha et al. found that Zika virus could damage the human placental barrier by inducing chronic placentitis, finding that viral proteins were detected in Hofbauer cells and some histiocytes in the intervillous spaces [33]. Bayer et al. found that the type III interferon (IFN), IFNλ1, produced by primary human trophoblasts could protect trophoblast and non-trophoblast cells from ZIKV infection [34]; this finding could be used for designing therapeutic strategies. 

### 2.4. The Mechanism of ZIKV-Mediated Brain Damage

Animal models have been used to investigate the link between ZIKV and brain damage. ZIKV infection has been found to cause placental damage and fetal demise in mice [35]. Cui et al. [36] used a mouse model and contemporary ZIKV strain to investigate the relationship between ZIKV infection and microcephaly. They found that ZIKV could replicate efficiently in embryonic mouse brain and cause cell-cycle arrest, apoptosis, and inhibition of neural precursor cell differentiation, leading to cortical thinning and microcephaly. Candidate flavirus entry receptor upregulation and genes associated with immune response, apoptosis, and microcephaly dysregulation were also found in the infected brains. Their model mimicked more closely the clinical findings in the human fetus including brains of smaller sizes, enlarged lateral ventricle, a thinner cortical plate and ventricular/subventricular zones in the infected mouse brains, providing evidence for a direct link between ZIKV infection and microcephaly. Rossi et al. used mice lacking interferon (IFN) alpha receptors to model ZIKV infection and found that ZIKV could be detected in the brain as soon as three days post-infection, with neurologic disease, such as tremors, detected six days post-infection [37]; these results indicated the neurotropic nature of the virus. Inoculation of pregnant mice with ZIKV was found to result in infected radial glia cells of the dorsal ventricular zone in the fetuses; because these cells are the primary neural progenitors responsible for cortex development, ZIKV infection resulted in reductions in the cavity of the lateral ventricles and cortex surface areas in fetal mice [38]. Although the mice were not an ideal model for describing the pathogenesis of ZIKV infection in humans, they provided an initial platform for the recognition of brain damage caused by ZIKV and evaluation of potential ZIKV antivirals and vaccines. Of course, further studies employing other animal models, such as nonhuman primates, are needed to confirm these findings. For the mechanism of human brain injury, some authors believe that ZIKV might reduce brain matter formation by affecting centrosome segregation, chromosomal stability, and autophagy [39]. Impairment of proper mitotic apparatus function is a possible mechanism by which ZIKV can exert teratogenic effects [40]. Using human-induced pluripotent stem cells, Garcez et al. explored the consequences of ZIKV infection in the first trimester of brain development with 3D culture models; these models demonstrated that ZIKV could lead to cell death in human neural stem cells, influence the formation of neurospheres and affect the growth of organoids [41]. In addition to the direct neurotrophic effects of ZIKV, its immunologic and autoimmune effects might be another important mechanism of brain damage. Anaya et al. [42] believed that gangliosides might play a role in the neurological complications associated with ZIKV infection. Gangliosides are abundant in the grey matter of the brain, and ganglioside expression influences neurogenesis, synaptogenesis, synaptic transmission, and cell proliferation. It is possible that during virus replication, ZIKV could induce an autoimmune response against gangliosides, resulting in an increased incidence of neurological complications and affecting fetal brain development. Dang et al. used human embryonic stem cell-derived cerebral organoids to investigate the relationship between ZIKV and microcephaly. They found that ZIKV could perturb cell fate and attenuate organoid growth by increasing activation of the innate immune receptor, Toll-like-Receptor 3 (TLR3), which could affect the apoptosis and neurogenesis pathways and play a key role in causing microcephaly [43]. Discernment of the immunologic mechanism of ZIKV is highly important because it can be used for vaccine development. Larocca et al. used susceptible mice to study a vaccine against a ZIKV outbreak strain from northeast Brazil and found that plasmid DNA and purified inactivated virus vaccines could afford complete protection against ZIKV [44]. Although it is difficult to extrapolate the protective effects of this vaccine directly from mice to humans, this study generated substantial optimism for the development of a safe and effective ZIKV vaccine for humans, and especially for pregnant women. Barba-Spaeth et al. found a subset of antibodies targeting a conformational epitope separated from patients of dengue fever also potently neutralize ZIKV. The crystal structure of these antibodies in complex with the envelope protein of ZIKV revealed the details of a conserved epitope, and the epitope might be related with virus maturation. This study provided the possibility of developing a universal vaccine protecting against both ZIKV and dengue virus infections [45]. In addition to the vaccine development, the research of antiviral drugs has achieved new progress. Xu et al. [46] screened over 6000 approved drugs and drug candidate compounds, by using ultra-high-throughput assays and human neural cells, to identify compounds that either protect against cell death in different neural cells or suppress ZIKV replication. Finally, a pan-caspase inhibitor, emricasan, 10 structurally unrelated inhibitors of cyclin-dependent kinases and a category B anthelmintic drug, Niclosamide, were proved to be effective. Their findings could have great implications for further ZIKV research and the development of anti- ZIKV therapeutics.

### 2.5. ZIKV Infection in the First Trimester of Pregnancy May Increase Susceptibility to Microcephaly

The relationship between viral infections during pregnancy and birth defects has long been recognized, and infection in the first trimester of pregnancy should be taken as a cause for increased concern [47]. Although it is possible that ZIKV could infect the fetus during any trimester of pregnancy [13], more and more authors believe that the greatest risk of microcephaly in newborns is associated with ZIKV infection occurring during the first trimester [25,48]. One study found that the number of microcephaly cases peaked after a lag of 30–33 weeks from the peak in acute exanthematous illness attributed to ZIKV, and they believed that this lag corresponded to the timing of infection in pregnant mothers during the first trimester [49]. Another study found that ZIKV incidence was most highly correlated with suspected microcephaly around week 17 of pregnancy and suspected severe microcephaly cases around week 14 of pregnancy in Brazil [50]. Using simple models, Cauchemez et al. retrospectively analyzed ZIKV and microcephaly data from French Polynesia between September 2013 and July 2015 to assess the periods of risk in pregnancy. According to that model, the baseline prevalence of microcephaly was two cases (95% CI: 0–8) per 10,000 neonates; however, the prevalence of microcephaly associated with ZIKV infection in the first trimester was 95 cases (95% CI: 34–191) per 10,000 neonates [51], which indicated that ZIKV infection during the first trimester of pregnancy might be associated with increased risk of microcephaly. These studies provided evidence of a temporal relationship between ZIKV infection in pregnant women during the first trimester and microcephaly. Fetuses infected during the first trimester might undergo pathologic changes during embryogenesis; however, infection during other trimesters might be associated with central nervous system abnormalities [19]. While pregnant women should avoid ZIKV infection during all trimesters, avoidance during the first trimester of pregnancy should be strongly emphasized.

## 3. Management, Prevention and Suggestions

No specific antiviral treatment or vaccine is currently available for ZIKV infections [3]; therefore, ZIKV infection treatment generally consists of supportive care, including rest, fluids, and use of analgesics and antipyretics [18]. For pregnant women, the most important way to avoid congenital ZIKV infection is to prevent maternal infection; this can be accomplished by either avoiding areas where ZIKV transmission is ongoing or avoiding mosquito bites [9,52]. The Centers for Disease Control and Prevention (CDC) recommends all pregnant women consider postponing travel to areas of ongoing ZIKV transmission if possible [53] and avoid unprotected sex with a partner who has recently traveled to a ZIKV endemic area [54]. Serial ultrasounds should be considered to monitor fetal anatomy and growth every three to four weeks in pregnant woman with laboratory evidence of ZIKV [14]. Serological tests and RT-PCR assays are recommended in infants whose mothers have had a risk of ZIKV exposure during pregnancy [55]. Further scientific studies are necessary for the prevention and control of ZIKV-related adverse outcomes. The development of vaccines and antiviral pharmaceuticals that are suitable for use in pregnant women and fetuses should be taken in the first place on the basis of existing research [44,45,46]. The development of rapid, scalable diagnostic tests is also needed because the current RT-PCR assay detects viral RNA and is therefore only effective for detection during the period of viremia, which may be relatively short. In addition, current serologic assays have considerable cross-reactivity with other pathogens such as Dengue virus which might be endemic in the same areas and cause symptoms similar to those of the ZIKV [23]. Besides, more evidence between ZIKV infection and microcephaly should be provided by case-control studies, animal model development, and other relative studies. 

## 4. Conclusions

In conclusion, there is increasing evidence suggesting that there is a causal link between ZIKV and microcephaly, especially ZIKV infection occurred in the first trimester of pregnancy. However, the mechanism by which ZIKV infection cause microcephaly needs more deep-in research.

## References

[1] Lanciotti R.S., Kosoy O.L., Laven J.J., Velez J.O., Lambert A.J., Johnson A.J., Stanfield S.M., Duffy M.R. (2008). Genetic and serologic properties of Zika virus associated with an epidemic, Yap State, Micronesia, 2007. Emerg. Infect. Dis..

[2] Campos G.S., Bandeira A.C., Sardi S.I. (2015). Zika virus outbreak, Bahia, Brazil. Emerg. Infect. Dis..

[3] Chan J.F., Choi G.K., Yip C.C., Cheng V.C., Yuen K.Y. (2016). Zika fever and congenital Zika syndrome: An unexpected emerging arboviral disease?. J. Infect..

[4] World Health Organization (WHO) Zika Virus, Updated 2016. http://www.who.int/mediacentre/factsheets/zika/en/.

[5] Duffy M.R., Chen T.H., Hancock W.T., Powers A.M., Kool J.L., Lanciotti R.S., Pretrick M., Marfel M., Holzbauer S., Dubray C. (2009). Zika virus outbreak on Yap Island, Federated States of Micronesia. N. Engl. J. Med..

[6] Cao-Lormeau V.M., Roche C., Teissier A., Robin E., Berry A.L., Mallet H.P., Sall A.A., Musso D. (2014). Zika virus, French Polynesia, South Pacific, 2013. Emerg. Infect Dis..

[7] Cardoso C.W., Paploski I.A., Kikuti M., Rodrigues M.S., Silva M.M., Campos G.S., Sardi S.I., Kitron U., Reis M.G., Ribeiro G.S. (2015). Outbreak of exanthematous illness associated with Zika, Chikungunya, and Dengue viruses, Salvador, Brazil. Emerg. Infect. Dis..

[8] World Health Organization (WHO) (2016). Zika Situation Report. http://apps.who.int/iris/bitstream/10665/250512/1/zikasitrep13Oct16-eng.pdf?ua=1.

[9] World Health Organization (2016). Zika Virus Microcephaly and Guillain-Barré Syndrome Situation Report. http://apps.who.int/iris/handle/10665/204454.

[10] World Health Organization (WHO) (2016). Women in the Context of Microcephaly and Zika Virus Disease. http://www.who.int/features/qa/zika-pregnancy/en/.

[11] Sahai I., Mochida G.H., Grabowski E.F., Caruso P.A. (2014). Case records of the Massachusetts General Hospital, Case 27–2014. A 10-month-old boy with microcephaly and episodic cyanosis. N. Engl. J. Med..

[12] Alvarado-Socarras J.L., Rodriguez-Morales A.J. (2016). Etiological agents of microcephaly: Implications for diagnosis during the current Zika virus epidemic. Ultrasound. Obstet. Gynecol..

[13] Oliveira Melo A.S., Malinger G., Ximenes R., Szejnfeld P.O., Alves Sampaio S., Bispo de Filippis A.M. (2016). Zika virus intrauterine infection causes fetal brain abnormality and microcephaly: Tip of the iceberg?. Ultrasound. Obstet. Gynecol..

[14] Burke R.M., Pandya P., Nastouli E., Gothard P. (2016). Zika virus infection during pregnancy: What, where, and why?. Br. J. Gen. Pract..

[15] Pan American Health Organization & World Health Organization (2015). Epidemiological Alert, Increase of Microcephaly in the Northeast of Brazil. http://www.paho.org/hq/index.php?option=com_docman&task=doc_view&Itemid=270&gid=32285&lang=en.

[16] Pan American Health Organization, World Health Organization (2015). Neurological Syndrome, Congenital Malformations, and Zika Virus Infection, Implications for Public Health in the Americas. http://www.paho.org/hq/index.php?option=com_docman&task=doc_view&Itemid=270&gid=32396&lang=en.

[17] World Health Organization (WHO) (2016). WHO Statement on the First Meeting of the International Health Regulations (2005) (IHR 2005) Emergency Committee on Zika Virus and Observed Increase in Neurological Disorders and Neonatal Malformations. http://www.who.int/mediacentre/news/statements/2016/1st-emergency-committee-zika/en/.

[18] Meaney-Delman D., Rasmussen S.A., Staples J.E., Oduyebo T., Ellington S.R., Petersen E.E., Fischer M., Jamieson D.J. (2016). Zika virus and pregnancy: What obstetric health care providers need to know. Obstet. Gynecol..

[19] Brasil P., Pereira J.P., Raja Gabaglia C., Damasceno L., Wakimoto M., Ribeiro Nogueira R.M., Carvalho de Sequeira P., Machado Sigueira A., Abreude Carvalho L.M., Cotrim da Cunha D. (2016). Zika virus infection in pregnant women in Rio de Janeiro—Preliminary report. N. Engl. J. Med..

[20] De Fatima Vasco Aragao M., van der Linden V., Brainer-Lima A.M., Coeli R.R., Rocha M.A., Sobral da Silva P., Durce Costa Gomes de Carvalho M., van der Linden A., Cesario de Holanda A., Valenca M.M. (2016). Clinical features and neuroimaging (CT and MRI) findings in presumed Zika virus related congenital infection and microcephaly: Retrospective case series study. BMJ.

[21] Marrs C., Olson G., Saade G., Hankins G., Wen T., Patel J., Weaver S. (2016). Zika virus and pregnancy: A review of the literature and clinical considerations. Am. J. Perinatol..

[22] Martines R.B., Bhatnagar J., Keating M.K., Silva-Flannery L., Muehlenbachs A., Gary J., Goldsmith C., Hale G., Ritter J., Rollin D. (2016). Notes from the field: Evidence of Zika virus infection in brain and placental tissues from two congenitally infected newborns and two fetal losses—Brazil, 2015. MMWR Morb. Mortal. Wkly. Rep..

[23] Rubin E.J., Greene M.F., Baden L.R. (2016). Zika virus and microcephaly. N. Engl. J. Med..

[24] Mlakar J., Korva M., Tul N., Popović M., Poljšak-Prijatelj M., Mraz J., Kolenc M., Resman Rus K., Vesnaver Vipotnik T., Fabjan Vodušek V. (2016). Zika virus associated with microcephaly. N. Engl. J. Med..

[25] Schuler-Faccini L., Ribeiro E.M., Feitosa I.M., Horovitz D.D., Cavalcanti D.P., Pessoa A., Dorigui M.J., Neri J.I., Neto J.M., Wanderley H.Y. (2016). Possible association between Zika virus infection and microcephaly—Brazil, 2015. MMWR Morb. Mortal. Wkly. Rep..

[26] Driggers R.W., Ho C.Y., Korhonen E.M., Kuivanen S., Jääskeläinen A.J., Smura T., Rosenberg A., Hill D.A., DeBiasi R.L., Vezina G. (2016). Zika virus infection with prolonged maternal viremia and fetal brain abnormalities. N. Engl. J. Med..

[27] Parra B., Lizarazo J., Jiménez-Arango J.A., Zea-Vera A.F., González-Manrique G., Vargas J., Angarita J.A., Zuñiga G., Lopez-Gonzalez R., Beltran C.L. (2016). Guillain-barré syndrome associated with Zika virus infection in Colombia. N. Engl. J. Med..

[28] Rasmussen S.A., Jamieson D.J., Honein M.A., Petersen L.R. (2016). Zika virus and birth defects—Reviewing the evidence for causality. N. Engl. J. Med..

[29] Besnard M., Lastere S., Teissier A., Cao-Lormeau V., Musso D. (2014). Evidence of perinatal transmission of Zika virus, French Polynesia, December 2013 and February 2014. Euro Surveill..

[30] Balm M.N., Lee C.K., Chiu L., Koay E.S., Tang J.W. (2012). A diagnostic polymerase chain reaction assay for Zika virus. J. Med. Virol..

[31] Petersen E., Wilson M.E., Touch S., McCloskey B., Mwaba P., Bates M., Dar O., Mattes F., Kidd M., Ippolito G. (2016). Rapid Spread of Zika Virus in The Americas—Implications for Public Health Preparedness for Mass Gatherings at the 2016 Brazil Olympic Games. Int. J. Infect. Dis..

[32] Adibi J.J., Marques E.T., Cartus A., Beigi R.H. (2016). Teratogenic effects of the Zika virus and the role of the placenta. Lancet.

[33] Noronha L.D., Zanluca C., Azevedo M.L., Luz K.G., Santos C.N. (2016). Zika virus damages the human placental barrier and presents marked fetal neurotropism. Mem. Inst. Oswaldo Cruz..

[34] Bayer A., Lennemann N.J., Ouyang Y., Bramley J.C., Morosky S., Marques E.T., Cherry S., Sadovsky Y., Coyne C.B. (2016). Type III interferons produced by human placental trophoblasts confer protection against Zika virus infection. Cell Host Microbe.

[35] Miner J.J., Cao B., Govero J., Smith A.M., Fernandez E., Cabrera O.H., Garber C., Noll M., Klein R.S., Noguchi K.K. (2016). Zika virus infection during pregnancy in mice causes placental damage and fetal demise. Cell.

[36] Li C., Xu D., Ye Q., Hong S., Jiang Y., Liu X., Zhang N., Shi L., Qin C.F., Xu Z. (2016). Zika virus disrupts neural progenitor development and leads to microcephaly in mice. Cell Stem Cell.

[37] Rossi S.L., Tesh R.B., Azar S.R., Muruato A.E., Hanley K.A., Auguste A.J., Langsjoen R.M., Paessler S., Vasilakis N., Weaver S.C. (2016). Characterization of a novel murine model to study Zika virus. Am. J. Trop. Med. Hyg..

[38] Wu K.Y., Zuo G.L., Li X.F., Ye Q., Deng Y.Q., Huang X.Y., Cao W.C., Qin C.F., Luo Z.G. (2016). Vertical transmission of Zika virus targeting the radial glial cells affects cortex development of offspring mice. Cell Res..

[39] Tetro J.A. (2016). Zika and microcephaly: Causation, correlation, or coincidence?. Microbes Infect..

[40] Bullerdiek J., Dotzauer A., Bauer I. (2016). The mitotic spindle: Linking teratogenic effects of Zika virus with human genetics?. Mol. Cytogenet..

[41] Garcez P.P., Loiola E.C., Madeiro da Costa R., Higa L.M., Trindade P., Delvecchio R., Nascimento J.M., Brinderiro R., Tanuri A., Rehen S.K. (2016). Zika virus impairs growth in human neurospheres and brain organoids. Science.

[42] Anaya J.M., Ramirez-Santana C., Salgado-Castaneda I., Chang C., Ansari A., Gershwin M.E. (2016). Zika virus and neurologic autoimmunity: The putative role of gangliosides. BMC Med..

[43] Dang J., Tiwari S.K., Lichinchi G., Qin Y., Patil V.S., Eroshkin A.M., Rana T.M. (2016). Zika virus depletes neural progenitors in human cerebral organoids through activation of the innate immune receptor TLR3. Cell Stem Cell.

[44] Larocca R.A., Abbink P., Peron J.P., Zanotto P.M., Lampietro M.J., Badamchi-Zadeh A., Boyd M., Ng’ang’a D., Kirilova M., Nityanandam R. (2016). Vaccine protection against Zika virus from Brazil. Nature.

[45] Barba-Spaeth G., Dejnirattisai W., Rouvinski A., Vaney M.C., Medits I., Sharma A., Simon-Lorière E., Sakuntabhai A., Cao-Lormeau V.M., Haouz A. (2016). Structural basis of potent Zika-dengue virus antibody cross-neutralization. Nature.

[46] Xu M., Lee E.M., Wen Z., Cheng Y., Huang W.K., Qian X., Tcw J., Kouznetsova J., Oqden S.C., Hammack C. (2016). Identification of small-molecule inhibitors of Zika virus infection and induced neural cell death via a drug repurposing screen. Nat. Med..

[47] Silasi M., Cardenas I., Kwon J.Y., Racicot K., Aldo P., Mor G. (2015). Viral infections during pregnancy. Am. J. Reprod. Immunol..

[48] Kleber de Oliveira W., Cortez-Escalante J., De Oliveira W.T., do Carmo G.M., Henriques C.M., Coelho G.E., Araújo de França G.V. (2016). Increase in reported prevalence of microcephaly in infants born to women living in areas with confirmed Zika virus transmission during the first trimester of pregnancy—Brazil, 2015. MMWR Morb. Mortal. Wkly. Rep..

[49] Paploski I.A., Prates A.P., Cardoso C.W., Kikuti M., Silva M.M., Waller L.A., Reis M.G., Kitron U., Ribeiro G.S. (2016). Time lags between exanthematous illness attributed to Zika virus, guillain-barré syndrome, and microcephaly, Salvador, Brazil. Emerg. Infect. Dis..

[50] Faria N.R., Azevedo Rdo S., Kraemer M.U., Souza R., Cunha M.S., Hill S.C., Thézé J., Bonsall M.B., Bowden T.A., Rissanen I. (2016). Zika virus in the Americas: Early epidemiological and genetic findings. Science.

[51] Cauchemez S., Besnard M., Bompard P., Dub T., Guillemette-Artur P., Eyrolle-Guignot D., Salje H., Van Kerkhove M.D., Abadie V., Garel C. (2016). Association between Zika virus and microcephaly in French Polynesia, 2013–2015: A retrospective study. Lancet.

[52] Hayes E.B. (2009). Zika virus outside Africa. Emerg. Infect. Dis..

[53] Centers for Disease Control and Prevention (CDC) (2016). Travel Health Notices. http://wwwnc.cdc.gov/travel/notices.

[54] Centers for Disease Control and Prevention (CDC) (2016). Interim Guidelines for Prevention of Sexual Transmission of Zika Virus—United States. http://www.cdc.gov/mmwr/volumes/65/wr/mm6505e1.htm.

[55] Flores M.S., Burgess T.H., Rajnik M. (2016). Zika virus: A primer for clinicians. Cleve Clin. J. Med..

